# Using intervention mapping to develop a work-related guidance tool for those affected by cancer

**DOI:** 10.1186/1471-2458-13-6

**Published:** 2013-01-05

**Authors:** Fehmidah Munir, Katryna Kalawsky, Deborah J Wallis, Emma Donaldson-Feilder

**Affiliations:** 1 School of Sport, Exercise & Health Sciences, Loughborough University, Loughborough, Leicestershire LE11 3TU, UK; 2 Affinity Health at Work, 287 Mayall Road, London, SE24 0PQ, UK

**Keywords:** Return to work, Sickness absence, Cancer, Intervention mapping

## Abstract

**Background:**

Working-aged individuals diagnosed and treated for cancer require support and assistance to make decisions regarding work. However, healthcare professionals do not consider the work-related needs of patients and employers do not understand the full impact cancer can have upon the employee and their work. We therefore developed a work-related guidance tool for those diagnosed with cancer that enables them to take the lead in stimulating discussion with a range of different healthcare professionals, employers, employment agencies and support services. The tool facilitates discussions through a set of questions individuals can utilise to find solutions and minimise the impact cancer diagnosis, prognosis and treatment may have on their employment, sick leave and return to work outcomes. The objective of the present article is to describe the systematic development and content of the tool using Intervention Mapping Protocol (IMP).

**Methods:**

The study used the first five steps of the intervention mapping process to guide the development of the tool. A needs assessment identified the ‘gaps’ in information/advice received from healthcare professionals and other stakeholders. The intended outcomes and performance objectives for the tool were then identified followed by theory-based methods and an implementation plan. A draft of the tool was developed and subjected to a two-stage Delphi process with various stakeholders. The final tool was piloted with 38 individuals at various stages of the cancer journey.

**Results:**

The tool was designed to be a self-led tool that can be used by any person with a cancer diagnosis and working for most types of employers. The pilot study indicated that the tool was relevant and much needed.

**Conclusions:**

Intervention Mapping is a valuable protocol for designing complex guidance tools. The process and design of this particular tool can lend itself to other situations both occupational and more health-care based.

## Background

It has been estimated that around 1 in 3 people (33%) will develop cancer at some point in their lifetime [1]. The most commonly diagnosed cancers are prostate, lung, colorectal and stomach for men; and breast, cervical, colorectal, and lung for women [2]. Together, these cancers account for 40% of all cancers worldwide [2]. In the UK, around 90,000 people of working-age are diagnosed with cancer annually [3], a time when career and work-related issues play an important role in their lives. However, advances in early detection and treatment of cancers have led to improved prognosis with five-year survival rates for all cancer combined reaching 50% [1]. Therefore, a high proportion of those treated successfully are able to resume their lives, including work [4] and between 41–84% of those treated for cancer are reported to return to work following treatment [5]. Evidence suggests, however, that many of these individuals can experience new physical limitations as a result of their illness and treatment [3]. Changes to physical function and long term/latent treatment side effects such as fatigue, depression, pain, cognitive deficits, can lead to a poorer overall quality of life [6,7], particularly with regard to working life [8-11]. These long-term effects may cause impairments that delay or prevent individuals returning to work. A review by Menhert [12] found the average length of sick leave for a person treated for cancer to be 151 missed days from work [12]; and cancer is associated with longer sick leave than other chronic conditions [13]. Sickness absence is an important economic outcome as missed days from work is a cost to society as well as to the employer and employee. Many employees affected by cancer want to work not just for financial reasons, but for overall health, well-being and higher quality of life [14]. However, those continuing to work during treatment, or return to work following cancer treatment; are more likely to have poor work ability compared to those with other chronic condition [13,15]. In addition, some employees affected by cancer experience job discrimination and lack of support from managers and occupational health professionals [9].

Some individuals are unable to return to work (RTW) and are at high risk of unemployment [8]. This has financial implications as well as further long-term implications for their health and overall quality of life.

In response to the work-related needs of those affected by cancer, a number of non-UK based healthcare-led interventions have been reported. These include providing information, counselling or advice about work or work-related issues [16-22], learning self-management skills in striving toward personal goals such as work [22], vocational training, job search assistance [23] and high-intensity physical training [24]. However, in nearly all of these interventions, breast cancer was the most common diagnosis and few of the interventions involved a multi-disciplinary approach that included health care services, the workplace and/or the employer.

At present, RTW interventions are complex to design and implement. Furthermore, due to the use of different study designs and measures, it is difficult to adequately judge the RTW success and other work outcomes (for example, work ability) of these interventions [25]. RTW interventions are further complicated by the different healthcare settings, workplace settings and stakeholders that exist, each with their own distinctive environment and the potential to impact return to work [26], making it difficult to interpret their success.

Although a number of robust interventions are underway [27,28], a recent review suggests that there are few support services in the UK designed to help people remain in, or return to work after cancer [29]. Issues relating to return to work for those affected by cancer have been identified as a major area for improvement by the UK National Cancer Strategy [30]. To address this, we developed a work-related guidance tool for those diagnosed with cancer to help them manage effectively their work or the return to work process so that they can make a timely return to work, manage the impact of their cancer-related health on their work, and manage the impact of work conditions upon their cancer-related health. The need for such a tool was prompted by the knowledge that healthcare do not necessarily comprehend the work-related needs of patients [31] and employers do not understand the full impact conditions such as cancer can have upon the employee and their work [32]. In these circumstances, it is often the patients/employees who lose out on the help and support they need to be able to return to work and/or prevent work disability. Therefore, we envisaged that the tool would be a self-led intervention that would enable those who have been diagnosed with cancer to take the lead and identify their work-related capabilities and limitations in relation to their diagnosis, prognosis and treatment, in consultation and discussion with a range of different healthcare professionals, employers, employment agencies and support services. In this respect the tool can be used by individuals with most types of cancer and in most work situations including those considering retirement or a change of employment. In addition, the tool would help those affected by cancer find solutions to their work-related needs by providing structural guidance in seeking relevant information and support from appropriate stakeholders, thereby enhancing the exchange of information. Therefore, the primary aim of the work-related guidance tool was to help those with cancer reduce sick leave and possible work restrictions and prevent unemployment and work disability by identifying work adjustments or ways to manage work with regard to their cancer-related health.

An intervention mapping (IM) protocol [33] was used to design the tool and to ensure it was grounded in evidence and in theory. Whilst traditionally used to develop health promotion programmes, IM has also been used for designing interventions, particularly complex interventions such as RTW programmes [26,34]. This is because RTW interventions require a tailored and multi-factorial approach directed at various settings and stakeholders [26,35], and IM provides a structured systematic framework within which to develop, implement and evaluate an intervention. Although we are not implementing or designing an intervention *per se*, but designing a work-related guidance tool that may be used for intervention purposes, IM is well suited for this process. This is because IM has been used to develop similar intervention tools for other health conditions such as occupational health guidelines to prevent weight gain among employees [36].

A detailed overview of how IM was used to design the tool is the focus of this paper.

## Methods

IM is a stepwise approach for theory and evidence based development and implementation of interventions. It consists of the following six steps, each leading to a product that guides the next step [33]: 1) a needs assessment; 2) the Identification of outcomes, performance objectives and change objectives; 3) selecting theory-based methods and practical strategies; 4) developing program components and materials; 5) planning for program adoption, implementation, and sustainability; and 6) planning for evaluation. Although presented as steps, IM is flexible process which makes it possible to oscillate between steps as new perspectives are gained.

In our study, we used steps one to four (creation of a work-related guidance tool) to ensure the resource development was grounded in theory and evidence [33,37]. This was important since the desired outcome of the tool was its wide use by individuals with different cancer diagnosis employed in a wide range of employment settings. Therefore, the present paper focuses mainly on how steps one through to four of the IM procedure were used to develop the work-related guidance tool. Step 5 is outlined briefly in the context of planning for program adoption as well as evaluation by others.

### Step 1: Needs assessment

The first step of the intervention mapping process was to conduct a needs assessment. This initially involved holding discussions with a key stakeholder, Macmillan Cancer Support, who is involved in both RTW research and in supporting employees with cancer to return to work. The objective of the needs assessment was to establish the rationale for a work-related guidance tool and to create an overview of the possible content of the tool. This was a three-stage process that included: 1) a review of the existing literature regarding cancer and work; 2) a review of existing work-related guidance tools; and 3) collection of new data using focus group discussions. The purpose for such a guidance tool was identified through these meetings, focus groups and the literature review.

The focus of the literature review was to identify the employment and work-related issues in adult cancer patients and the existence of any work-related guidance tool. For the academic literature search we searched the databases PubMed, Medline, Web of Science, PsychInfo and Google Scholar, combining ‘cancer’ with each of the following terms: ‘employment/work review’, ‘employment’, work needs’, ‘work ability’, ‘work disability’, ‘work limitations’, ‘work adjustments’, ‘return to work’, ‘work issues’, ‘work changes’, ‘sickness presenteeism’, ‘work problems’, ‘work restrictions’, ‘work difficulties’, ‘sickness absence’, and ‘sick leave’. Reference lists of the relevant articles were also searched. To identify the existence of any work-related guidance tools, we used the search terms: ‘tool’, ‘measure’, ‘questionnaire’, ‘scale’, ‘instrument’ ‘assessment’, and combined each of these with ‘patient self-management’, ‘cancer self-management’, ‘cancer, work and self-management’. The reference lists of identified articles were searched manually for existing measures or tools. Additionally, organisations and specialists in the area of rehabilitation were contacted by the research team to further identify self-management tools that focused on return to work and work ability. All searches were restricted to between January 2000 and October 2010.

The lay literature search focused on websites and pamphlets written and published by cancer charities (for example, Macmillan Cancer Support, Bowel Cancer UK) and intended for the general public. Appropriate websites were identified via the Google search engine. Any external links to other websites were also searched but only if they were tailored towards financial, legal and employment matters in the UK (for example, the Department for Work and Pensions, Jobcentre Plus).

Finally, focus groups were conducted with those who were being treated for cancer (with curative intent) or had recovered from cancer. The purpose of the focus groups was to explore additional gaps from the literature review in patient knowledge and gaps in information/advice received from healthcare professionals (and other stakeholders groups) with regard to cancer and work issues. The focus groups also informed the content and structure of the tool.

#### Focus group participants and procedure

Participants for the focus groups were recruited from various Macmillan Cancer Support sources (for example, Macmillan Cancer Voices Network, Macmillan Online Community, Macmillan Face book Group, and Macmillan e-newsletters/bulletins), cancer support groups and media press releases (including local newspaper and radio advertisements). The following inclusion criteria were applied for participation: 1) to be aged between 18–65 years; 2) to have been employed at the time of diagnosis; and 3) to have been diagnosed with cancer no more than five years ago. The criterion of five years was chosen to provide a reasonable time frame in which to expect return to work, to enhance accurate recall of recent work issues and to gain knowledge about the impact of late and/or latent treatment side effects on work.

Those still wishing to participate contacted the researcher directly and completed a recruitment questionnaire (either in person or over the telephone) to ensure that they met participation criteria. Those identified as being suitable for study enrolment were asked to provide written informed consent. The participants who took part in the focus group were selected and considered as ‘experts’ based on their knowledge and experience of living with a diagnosis of cancer. The study was approved by Loughborough University’s Ethical Advisory Committee. As we were not intending to recruit patients directly from NHS Trusts or run a healthcare intervention, NHS ethical approval was not required.

Thirteen participants (aged 34–63) were recruited and took part in one of two focus groups at hired venues. As the majority (n=10) were female, individual interviews were conducted with six males (aged 37–65) with or recovering from cancer, in order to prevent gender bias amongst the study findings. Within the whole sample, there was a variety of cancer types and occupations see Table 1 for a summary. The overall organisation and moderation of the focus groups and interviews was co-ordinated and managed by the same researcher (KEAK). An interview schedule was used to provide a degree of consistency and structure to the process. Questions to facilitate discussion were open ended around the following topics:

a) Existing information surrounding cancer and work issues

b) Information needs surrounding vocational (and other) rehabilitation support

c) Financial implications of cancer

d) The impact of long term and/or latent side effects of treatment on work

e) Psychological and emotional needs at work

f) Training and new skills needed to support return to work

g) Dealing with health insurers

h) The interview/recruitment process (i.e. with a prospective employer)

**Table 1:** Participants characteristics from focus group and interviews

**Participant**	**Age**	**Gender**	**Time since diagnoses**	**Cancer type**	**Treatment**	**Job type**	**Work status**	**Self-employed**
1	34	Male	8 months	Non-Hodgkin’s lymphoma	S,C	Non-Manual	Returned full time	No
2	42	Female	14 months	Breast	S	Non-Manual	Returned full time	Yes
3	36	Female	32 months	Mixofibrosarcoma of the jaw	S	Non-Manual	Job seeking	No
4	60	Female	11 months	Breast	S,R	Manual	Returned full time	No
5	45	Female	19 months	Appendix	S,C	Manual	Returned full time	Yes
6	48	Female	35 months	Breast	S,C,R	Non-Manual	Returned full time	No
7	54	Female	36 months	Breast	S, C,R	Non-Manual	Returned full time	No
8	53	Female	18 months	Hodgkins lymphoma	C	Non-Manual	Returned part time	No
9	44	Female	14 months	Breast	S,C	Non-Manual	Returned part time	No
10	46	Male	13 months	Myeloma Bence Jones	Unknown	Manual	Sick leave	Yes
11	43	Female	17 months	Skin	S	Non-Manual	Returned full time	N/A
12	63	Male	32 months	Bowel	S,C	Not applicable	Retired	No
13	46	Female	55 months	Breast	S,C	Non-Manual	Returned full time	No

Key: S = Surgery; C= Chemotherapy; R= Radiotherapy.

All participants were provided with the interview questions before the start of the focus group or interview to optimise accurate recall. In addition, prompts were used as appropriate to gain clarification and to encourage respondents to elaborate on relevant topics. The focus group discussions and interviews were digitally recorded and transcribed verbatim. The duration of the focus groups and interviews was approximately one hour. Data were analysed using thematic analysis [38]. The reliability of the analysis was ensured through a systematic review of the data by all members of the research team. Following agreement on the themes identified, a table of themes was drawn up and the text passages were coded accordingly.

### Step 2: Identification of intended outcomes and performance objectives

The purpose of Step 2 of the IM procedure was to specify who and what would change as a result of the work-related guidance tool. First, we defined the desired behavioural and environmental outcomes for the target group that need to occur in order to affect the determinants of the overall behavioural objectives identified in step 1. The overall desired behavioural outcome was for those with/recovering from cancer to identify possible solutions to work-related needs (such as work adjustments and ways to manage work). Specific desired outcomes were based around this. Second, performance objectives for each of the desired behavioural outcomes were specified. These are a step-by-step description of what the participants will do or how an environmental condition will be modified (including who will create the change) [33]. For example, if a desired behavioural outcome is for those with/recovering from cancer to make informed decisions related to their cancer and work issues, then the performance objective for that outcome would be for participants to identify appropriate support and resources. Performance objectives were then examined in light of determinants of behaviour and the environment to produce change objective statements. Change objectives are the most immediate targets of an intervention [33] and are specified in terms of who or what needs to change and/or be learned in order to affect the performance objective. As one of the aims of our intervention was to develop a tool to be used by those with/recovering from cancer but that would be able to influence relevant stakeholders (e.g. employers, healthcare professionals) performance objectives and change objectives were also developed in relation to this. In order to develop change objectives, each performance objective is scrutinised separately and appropriate theoretical determinants identified. For example, if a performance objective is for individuals to ‘self-manage’ their use of the work-related guidance tool, an appropriate theoretical determinant may be self-efficacy [39].

### Step 3: Selecting theory-based methods and practical strategies

The third step of the intervention mapping process involved identifying suitable theoretical methods to change behaviour and translating these into practical strategies. Bartholomew [33] states that the goal of step 3 is to use a conceptual model or theory (for example, socio-cognitive theory) to guide the identification of appropriate intervention methods and delivery strategies related to the objectives stated in step 2. In this step, using evidence from the literature review and focus groups, we identified appropriate theoretical methods and strategies for our stated objectives.

### Step 4: Developing program components and materials

In step four, a description of the scope and content of the tool is outlined. The steering group (members of the National Cancer Survivorship initiative and Macmillan Cancer Support) and expert stakeholders provided guidance regarding the scope and content of the tool. A feasibility study was carried out to test the work-related guidance tool and to ensure it met the change objectives and practical strategies identified in Step 3.

### Step 5: Planning for programme adoption and implementation and step 6: creating an evaluation plan

In step five, a plan for programme adoption and implementation was outlined; and in step six, a plan for evaluation was generated. Both step five and six are not in the scope of the current paper; they will only briefly be discussed in the results.

## Results

### Step 1: Needs assessment

#### Literature review

The academic literature is focused largely on return to work rates and risk factors known to hinder successful work resumption and/or work ability (for example, age, educational attainment, cancer stage, treatment and occupation type). Other studies have focused on the workplace concerns of those with/recovering from cancer. For example, apprehension over financial security [40]. A range of studies, however, have identified what could be done to facilitate return to work and work ability [9,31-47]. These have reported that flexible working hours (for example, to attend medical appointments) and adjusted working arrangements (for example, to start their shift later in the day) can improve return to work and work ability [9,45-47]. This was also supported by the lay literature, which frequently reported that employees have the right to request modifications to their working pattern or ask for other “reasonable” adjustments [48]. Other potential needs identified were related to information about the financial implications and the potential side effects (i.e. physical and emotional consequences) of cancer and its treatment on work [46]. A review of return-to-work intervention studies for breast cancer survivors found exercise and counselling associated with return to work [49]. Another review on return-to-work interventions of all cancer types reported that encouragement, education or advice about work or work-related topics, vocational training (for example, learning self-management skills) and work adjustments had some evidence of effectiveness for return-to-work outcomes [25]. In sum, the working needs of those with or recovering from cancer were categorised into three key topic areas:

1) Health

2) Work

3) Finance

For the work-related guidance tool review, few relevant articles were identified. This was because most existing tools were too specific (i.e. in terms of different health conditions) and did not focus on work-related issues. Similarly, no relevant tools were identified by rehabilitation experts and organisations.

#### Focus group and interview data

Table 1 summarises the participant characteristics. Data from the two focus groups and the interviews suggested three main themes relating to both positive and negative work experiences of those with or recovering from cancer, and their work-related needs. These work-related needs were identified as the most important information or support required by participants to make an informed choice about their work and return to work. The most important work-related needs of the participants are summarised in Table 2 along with a number of sub-themes related to each main theme. The main themes identified were: health-related issues and their impact on work (for example, information on how the ‘fit note’ process is managed); workplace information and support (for example, workplace accommodations and flexible working hours to accommodate medical appointments and the side effects of treatment); and financial-related issues following diagnosis and treatment (for example, clarification on insurance policies and additional benefits).

**Table 2:** Summary of themes related to work-related needs and guidance

**Main theme**	**Sub-theme**
**Work**-place information and support	· Workplace accommodations
	· Awareness of new information
	· Contact with co-workers
	· New employment disclosure
	Company policies
**Health**-related issues on work	· Information provision
**Finance**-related issues following diagnosis and treatment	· Information provision

Participants discussed a range of factors for each main theme. For example, for work information and support they felt that, in order to aid the transition back to work, it was important to be updated on changes that may have occurred in the workplace during their absence (i.e. awareness of new information). They also discussed the importance of maintaining contact with co-workers during sickness absence. Some participants expressed concern over ambiguity surrounding what information their previous/current employer was allowed to disclose to a future employer and felt that more information was required regarding this matter (new employment disclosure). It was also important to participants that their company policies surrounding sickness absence, sick leave entitlement, sick pay arrangements and how much notice is required before returning to work were made clearer and more easily available. With regard to health, participants highlighted a need for better information provision from various parties regarding the possible impact of cancer and its treatment on work. They expressed particular concerns regarding information relating to understanding the nature of potential side effects (i.e. physical and/or psychological) and being enlightened as to when they may interfere with work (information provision). Finally, many participants indicated that they needed greater clarification regarding financial matters associated with cancer and its treatment. Some participants felt that there was a lot of uncertainty regarding how they were covered by their insurance policies and others felt that information was limited in terms of obtaining additional benefits, for example, to cover additional travel costs.

### Step 2: Identification of intended outcomes and performance objectives

Based on the findings of the focus groups and literature review, the overall desired outcome for the intervention tool was defined as ‘a self-management of work-related decisions’. Due to the differing occupations and work patterns of the intended users, it was clear that the guidance tool needed to be designed so that there was relevance to almost all employee groups. Therefore, the three key target behaviours for those with/recovering from cancer were 1) to take control over work-related issues; 2) to make informed decisions related to cancer and work issues; and 3) to enhance their return to work and/or work ability. As involvement of relevant stakeholders was vital for those with/recovering from cancer in achieving the three behaviour targets, we identified that we required those with/recovering from cancer to clearly communicate their needs to the stakeholders and to engage them in supporting their needs. It was therefore decided that the tool would need to consist of relevant questions that those with or recovering from cancer could ask their employer in order to promote dialogue. This way, an employer could provide a response (in the form of information, support or action) relevant to the individual, their circumstances and their job and work setting. The needs assessment also identified health care providers (general practitioners, oncologists, oncology nurses), unions, occupational health services, charities and benefit advisors as important stakeholders for employees making decisions about work, especially for those self-employed or those not returning back to work/back to the same workplace. Therefore, the work-related guidance tool would also contain relevant questions for such stakeholders.

The next stage of the intervention mapping process was to specify the performance objectives. Using the above information, the research team listed the steps that would need to be taken in order to achieve the overall intended outcome. Next, the performance objectives for each of the desired behavioural outcomes were identified. These can be found in Table 3. To create a matrix of ‘change objectives’, the main personal determinants (factors within the individual and in their direct control) and external determinants (factors that can directly influence the health behaviour or environmental conditions) of behaviour change for each performance objective were first operationalised. The three categories of determinants from the Theory of Planned Behaviour [50] were considered appropriate and were targeted. These are: attitude (for example, how positive the individual is to ask for support); social influence (for example. the subjective norms and social norms of receiving support from others); and self-efficacy (for example how confident the individual is to approach relevant stakeholders for support). Intention and knowledge were also identified as important determinants of the performance objectives. The tool aims to influence all these determinants but especially self-efficacy and knowledge. Self-management intervention programmes have shown that changes in self-efficacy (a determinant based on Bandura’s Social Cognitive Theory [51] are associated with changes in behaviour and health status for a range of chronic conditions e.g. [52]. The final step is to create a matrix with all the key information by mapping performance objectives (row) against determinants (column headings). The cells are then filled with what the target group should do and/or know and what should change in the environment in order for there to be a positive impact on each determinant so that the performance objective can be achieved.

**Table 3:** Matrix for change objectives

**Performance objectives**	**Personal determinants**				**External determinants**
	**Intention**	**Self-efficacy**	**Attitude**	**Knowledge**	**Social influence**
1. Communicate with relevant stakeholders	Formulate and implement intention to discuss work-related issues and solutions for work	Demonstrate ability to approach relevant stakeholders	Believe that if you communicate with stakeholders, it will result in provision of information and support	Understands purpose of the tool and how to use it	Perceive stakeholders are discussing work-related issues and solutions for work with others; manage expectation of stakeholders about yourself and your current level of work ability
2. Request information and support		Shows confidence to ask the questions on the tool to relevant stakeholders	Accept that there will be benefits and positive feelings in requesting information and support	Identify from the tool the information, support and resources that is personally required; Identify which questions to ask on tool and to whom	
3. Assess adequacy of information, resources and support related to work/return to work decisions about work		Shows confidence in assessing personal and environmental situation in relation to information, support and resources offered by stakeholders		Know which information, support or resources offered by stakeholders is beneficial to your own personal situation	
4. Make decisions about work based on information and support utilised		Demonstrate following an action plan	Believe that there will be benefits in the decisions made	Know how to use acquired information to make informed decisions about work and return to work	
5. Use the work-related guidance tool as often as required through the cancer journey	Increase motivation to use the tool when needed; and increase motivation to monitor current situation	Demonstrate ability to monitor current situation with regard to health and work-related issues		Know when to use the tool	

### Step 3: Selecting a theory-based method and practical strategies

Given the identified outcomes and objectives of the tool, empowerment was selected as the theoretical framework best suited to underpin tool design, in order to influence users’ self-efficacy and knowledge. Empowerment has been defined as a means by which individuals gain a sense of control over their lives, particularly with regard to decision making [53]. Therefore, empowerment is a potential mechanism for increasing self-efficacy, as it enables an individual to feel competent and confident in their ability to perform self-management behaviour [54,55]. Furthermore, empowered patients are in a better position to gather knowledge and having knowledge is likely to increase empowerment. The objectives of empowerment interventions used in the workplace focus on skills and behaviour change by improving, for example, employees’ action planning activities and self-efficacy. Self-management programmes for employees also focus on similar strategies see [56]. The research team subsequently identified practical strategies that are thought to influence the theoretical determinants using empowerment theory as well as other appropriate theoretical methods [33]. Theoretical methods and practical strategies are specified in Table 4.

**Table 4:** Theoretical methods and practical strategies

**Performance objectives**	**Behaviour change techniques**	**Strategies**
1. Communicate with relevant stakeholders	Implementation intentions Verbal persuasion (SCT)	Individuals are given the tool by Macmillan Cancer Support staff and/or other support services or they can download from the Macmillan website. One-to-one discussion with Macmillan Cancer Support staff and information available via their website will be given to encourage implementation of intentions (i.e. to discuss work-related issues). A questionnaire format would be used to enhance the usability of the tool. Questions in the tool are tailored to encourage users to take a pro-active approach to obtain relevant information related to potential cancer and work issues (i.e. ‘empowering’ questions).
2. Request information and support	Goal setting	Through questions posed in the tool, individuals can activate social support at work and from other key stakeholders. Questions will consist of goal setting questions e.g. How can we work together to make decisions about any changes to my job role and description? Each question in the tool will clearly indicate which stakeholder(s) are important to ask.
	Individualisation (TM) Active processing of information	The guidelines in the tool will provide a clear journey for users to follow, but be flexible enough for them to choose which section applies to them. Written guidelines in the tool will emphasise the flexibility for the individual in choosing one or more important stakeholder to ask the question (from those indicated; and where more than one stakeholder has been identified) as relevant to their needs.
	Guided practice (SCT) and Enactment (SCT)	The tool is portable in design so that users can take it with them to meetings with relevant stakeholders. Individuals can practice asking the questions beforehand. Using the tool frequently throughout the cancer journey should result in mastery experience.
3. Assess adequacy of information, resources and support related to work/return to work decisions about work	Decisional balance (SCT) Feedback (ToL)	Individuals write down the feedback given by stakeholders in order to identify obstacles and solutions and to assess which information given, support or resources offered will be most beneficial to them.
4. Make decisions about work based on information and support utilised	Persuasion Feedback (ToL)	Individuals view and discuss their options and/or decisions with stakeholders as well as with significant others.
5. Use the work-related guidance tool as often as required through the cancer journey	Implementation intentions Goal setting Shifting focus (TPB)	Individuals use the tool throughout their cancer journey. They re-evaluate their current situation and their needs, and shift their focus by using other questions in the tool to receive appropriate information and support.

Key: SCT = Social Cognitive Theory; TM= Transtheoretical model; TPB= Theory of Planned Behaviour; ToL= Theories of Learning.

### Step 4: Developing program components and materials

The first phase in Step 4 was to decide the scope of the work-related guidance tool. To determine the structure of the work-related guidance tool, we carried out another literature review to identify existing empowerment tools and evaluate their content. The academic and grey literature were searched using a similar search strategy for identifying self-management tools to that outlined in Step 1. The results from this search identified a large degree of variability among existing measures of empowerment. For example, some tools require respondents to indicate their level of agreement towards a list of statements that draw upon concepts such as self-efficacy, perceived control, self-esteem and a sense of responsibility [57,58]. Other tools comprise a list of questions that aim to encourage patients to communicate with various healthcare professionals [59]. Therefore, whilst some measures are designed to determine an individual’s current level of empowerment, others are designed to enhance perceptions of empowerment. Since a key aim of our tool was to empower those with or recovering from cancer to effectively manage their work or the return to work process, it seemed appropriate to develop a tool that consisted of ‘empowering questions’ to encourage individuals to become active communicators with key stakeholders such as healthcare professionals, employers and employment agencies. Using the information collated from the above steps of the intervention mapping protocol, the first draft of the tool was developed. It consisted of 43 questions that were divided into one of four categories to represent the stages of the cancer journey in relation to work:

1) Initial work issues and absence from work

2) Preparing to return to work

3) Returning to work

4) Not returning to work

Within each of these categories, the questions were organised into three broad themes:

1) Health

2) Finance

3) Work

For each question in the tool, a list of seven stakeholders was provided (e.g. Oncology team, General Practitioner, Occupational Health) in order that respondents could state to whom each question should be asked. A sample of the tool is presented in Table 5.

**Table 5:** Sample of the tool content (final version)

**When?**	**Topic area**	**What do I ask?**	Who do I ask? **							
			**Oncology team**	**GP**	**Occupational Health**	**Line manager**	**Human Resources**	**Advisory Services**	**Charity/ support group**	**Union**
**Initial work issues and absence**	**Health**	How much time will I need to take off work for each of my treatments (e.g. surgery, chemotherapy, radiotherapy, hormone therapy etc.)?	**✓**	✓	✓					
		How will my fit note certification be managed? (NB: In 2010 the fit note replaced the sick note)	✓	**✓**	✓	✓	✓			
		Which treatment side effects are most likely to interfere with my work? When will these side effects occur and how long will they last?	**✓**	**✓**	**✓**					
		What support services are available to me (e.g. counselling, employee assistance programmes, occupational therapy, vocational rehabilitation, etc.)?	✓	✓	✓	✓	✓	✓	✓	✓
	**Finance**	If I were unable to work, what benefits would be available to me (e.g. statutory sick pay, employment and support allowance, disability living allowance, company sick pay, etc.) and how can I access this help?					✓	**✓**	✓	✓
		If I’m unable to work, what insurance and payment protection policies might I have that are relevant?					✓	**✓**	✓	
		For how long can I go on claiming benefits?					✓	**✓**	✓	
	**Work**	Where can I find information on the company’s policies relevant to my situation (e.g. absence management, occupational health, sick pay, pension scheme, etc.)?			✓	**✓**	**✓**			✓
		Will I have to use my annual leave entitlement instead of taking sick leave?				**✓**	**✓**			✓
		How soon can you confirm my sick pay arrangements?				✓	**✓**			
		What do we need to do to ensure that my job will be secure if I take time off work?			✓	**✓**	**✓**			✓

Next, it was necessary to determine precisely how the tool as should be delivered. An important element of the tool was that it should be self-led by those with/recovering from cancer and could be used throughout their cancer journey as appropriate. The tool could be delivered to the target group by making it available to them at oncology clinics by oncology nurses and/or oncologists. This was a realistic approach as oncology clinics should be providing work-related information and support to cancer patients as part of their care (see National Cancer Survivorship Initiative website http://www.ncsi.org.uk). The tool could also be delivered by Macmillan Cancer Support nurses and support groups and on the Macmillan Cancer Support website as well as other websites such as on the National Cancer Survivorship Initiative website. As the premise for the tool was that not only should it be self-led requiring minimal input from experts, it should also be applicable to a variety of different cancer types, occupations and work settings, the above delivery strategies were pragmatic. The guidance for using the tool was then written taking into account the steps above.

To ensure that the content of the tool met the intended change objectives, that it would empower the target audience, could be used without expert delivery, and that there was a clear rationale regarding to whom each question should be directed, a Delphi study was carried out. The Delphi method is a systematic technique which aims to engage a large number of experts (i.e. those who specialise in a particular field of interest or who have knowledge about a specific subject) in a process to obtain consensus of opinion or judgment on a topic where the required information is incomplete or scarcely available [60]. It is an iterative process based on the results of several questionnaire rating rounds. Responses are analysed and statistically summarised (usually into medians and upper and lower quartiles) and presented to the expert panel for further consideration [61]. In the present study, a two-round Delphi consensus method was used and the following expert groups were identified for this process: those with/recovering from cancer and in various stages of their cancer journey/return-to-work process; healthcare professionals relevant to cancer patients (e.g. General Practitioners, oncologists, nurses); occupational health staff, employers and employer representatives (e.g. human resources, line managers, trade unions); charities (e.g. Macmillan Cancer Support); benefit advisors (e.g. Job Centre Plus); and researchers with expertise in cancer and return to work issues (both national and international experts). These experts were recruited from a variety of different sources. Ethical approval was obtained from Loughborough University.

One hundred and seventy-two ‘experts’ took part in the Delphi study. Participant characteristic are presented in Table 6. This was conducted online where each participant was asked to rate each question (on a nine-point Likert scale) the degree to which they thought that the question posed would help the target group make decisions related to work and well-being (1 = not at all, 9 = a great deal); and to whom the question should be asked (e.g. Oncology Team, Occupational Health, Line Manager, Human Resources) (multiple answers were permitted). Open textboxes were also provided for feedback on each question or section. Following feedback, one hundred and thirty nine participants completed the second round of the Delphi, responding to the questions that did not reach consensus in the first round. Data were analysed further to assess consensus between the experts. This resulted in 40 questions for the tool and a range of agreed stakeholders relevant for responding to each question. A feasibility study was then carried out with an independent group of participants who had or were recovering from cancer (n=38) and who were at various stages of the cancer journey. The purpose of the feasibility study was to assess whether the tool met the change objectives and potential strategies. Again, the participants were recruited from a variety of sources (excluding the National Health Service) such as local cancer support groups and networks, and through an advertisement placed in the local paper. Those expressing an interest were asked to complete a consent form and inclusion criteria form (must have been in employment at time of diagnosis; no history of psychiatric disorders). Those meeting the inclusion criteria were then sent a link to an online survey which asked participants to rate their self-efficacy and perceptions of empowerment regarding work decisions. After they had completed the survey, they were sent a hard copy of the tool via postal service. The tool was tested over a six week period after which participants were asked to complete the same online survey again and additional questions on their experience of using the tool, including how many questions they used, what responses they obtained and how useful each question was for their needs (process evaluation). Analyses of the data suggest that levels of self-efficacy and feelings of empowerment improved over the six week period. However, there was no control group and therefore these results must be interpreted with caution. Findings from the feasibility study and its evaluation will be presented in a separate paper.

**Table 6:** Delphi experts for round one and two of the Delphi process

**Stakeholder**	**Delphi round one (n)**	**Delphi round two (n)**
Individual with cancer	68	57
Oncology Team	13	12
GP	5	5
Other Healthcare Professional	16	8
Occupational Health	15	12
Line Manager	5	4
Human Resources	19	15
Advisory Services	3	2
Charity/Support Group	2	1
Union Advisor	3	3
Researcher	23	20
Total	**172**	**139**

### Step 5: Planning for program adoption and implementation

Once the tool was finalised, the next stage was to create a plan for the adoption and implementation of the tool amongst the target group. This meant developing an implementation plan and training for healthcare professionals, support groups and employers who could direct those with/recovering from cancer to the tool. This is currently being led by Macmillan Cancer Support who have adopted and implemented the following strategies: 1) 10,000 printed copies of the tool in 2011 distributed to Macmillan Cancer Support services, vocational rehabilitation support services and other support services for dissemination to patients and service users. Printing, distribution and dissemination of the tool is on-going; 2) provision of downloadable PDF version of the tool through their website and through the National Cancer Survivorship website; 3) printed copies of the tool are made available in a ‘toolbox’ that employers can order for free from the Macmillan website 4) training HR and line managers in how to respond to the use of the tool and how to provide support to employees diagnosed with cancer. Through the Department of Health and employer organisations such as Chartered Institute for Personnel Development (CIPD) Macmillan have raised awareness of the tool and mobilised human resources personnel, line managers and employers for training on the usage of the tool. The tool has also been evaluated by an independent research team as part of the evaluation of the cancer vocational rehabilitation pilots (by the University College of London on behalf of Macmillan Cancer Support and the National Cancer Survivorship Initiative).

### Step 6: Creating an evaluation plan

The effectiveness of the tool will be evaluated in a randomised control trial (RCT). Patients who have been diagnosed with cancer and are in employment at the time of diagnosis will be invited to participate via oncology clinics and will be randomised into a control (no intervention) group and a intervention group who will be asked to use the tool for twelve months. The primary outcome measure is defined as: duration of sick leave in calendar from first day of sick leave to full or partial return to work. Secondary outcome measures include quality of working life; general quality of life; psychological well-being; job self-efficacy and perceptions of empowerment. Questions to evaluate behavioural determinants will also be included. Assessments will take place at baseline, and after three, six, nine and twelve months. A process evaluation will also be conducted to assess satisfaction and utility of the tool. A power calculation is yet to be determined as this may need to be conducted separately for different types of cancer. It might be that we conduct an RCT in only four (or fewer) types of common cancers. Ethical approval will be sought from the NHS Trust ethics committee. Details about the evaluation of the tool will be presented in a separate paper.

## Discussion

In this report, we have described the development of a work-related guidance tool using intervention mapping. To our knowledge, this is the first study to use IM to develop a tool as a self-led intervention itself, rather than an intervention *programme* per se. IM has been proven to be a useful protocol for developing not only health promotion programmes but also RTW programmes [34,56,62]. We found IM to be a useful planning template as it enabled a tool to be developed that is theoretically grounded and evidence-based, with independent, systematic involvement from key experts (the Delphi study and feasibility study) to ensure the tool met all its objectives. This is one of the key strengths of using IM; by using conceptual models, we were able to construct a work-related guidance tool that is pragmatic and credibly tailored to the needs of a specific population. A key strength of our work-related guidance tool is that it is designed to be a self-led intervention that is underpinned by a theoretical framework. Therefore, there is no training required for those with/recovering from cancer in using the tool. This means that very little input is necessary by health care professionals, who already have a demanding workload. In terms of raising awareness of the tool among healthcare professionals, employers and those with/recovering from cancer, as outlined in step five, much of this work is currently underway by Macmillan Cancer Support. Although the number of RTW interventions is increasing, none have been based on a self-led tool that empowers the patients by encouraging them to take the lead and identify their work-related capabilities and limitations in relation to their diagnosis, prognosis and treatment. In addition, the tool promotes consultation and discussion with a range of different healthcare professionals, employers, employment agencies and support services who are all involved in the RTW of an individual diagnosed and treated for cancer. This is a unique aspect of our tool which is made distinctive by the involvement of these key stakeholders in confirming the appropriateness and relevance of each question in the tool in the Delphi consensus process. To our knowledge, no similar technique has been used for developing RTW interventions for those affected by cancer.

Our literature review and focus group data revealed these stakeholders to play a key role in facilitating or creating an obstacle for making a timely return to work and in obtaining appropriate workplace support. Our focus group data further reflect the current literature on the obstacles and facilitators to workplace accommodations [32]. It is envisaged that the tool will address some of the problems raised in the literature with regard to reducing sickness absence, minimising the risk of unnecessary unemployment and financial problems; job discrimination and lack of emotional and practical support from line managers, employers, occupational health services and healthcare services [4,32,46].

A possible limitation to using IM is that it is time consuming. The process described in this paper took nine months to complete. It required a full time researcher and six weeks full-time work by the research team. This is a similar experience reported by other researchers using IM [56,62]. Due to both funding and time constraints, it was not possible to create a complete description of the adoption, implementation (step 5) and evaluation plan for the work-related guidance tool. Although Macmillan Cancer Support and the NCSI have developed an adoption plan for the tool, this may not be rigorously assessed. This is a particular concern as the contribution of IM in using theoretical knowledge and strategies to underpin the tool may not be evaluated and documented. Another issue of concern is whether we are able to test the tool in an RCT as Macmillan Cancer Support and the NCSI have already made the tool available through channels. Therefore, it may be difficult to recruit a control group without potential exposure to the tool and contamination by such exposure. These issues will have to be resolved. A possible weakness of the study is that although we included a wide range of stakeholders from a variety of backgrounds and experiences, the employers involved were mainly from medium to large organisations. Therefore the tool may not capture the perspectives of small organisations. However, a representative proportion of our participants with/recovering from cancer either worked for a small organisation or were self-employed and therefore their perspectives have contributed to the development and refinement of the tool.

## Conclusions

In this study we describe the development of a self-management work-related guidance tool for those with/recovering from cancer, using the Intervention Mapping framework. The tool has already been implemented by Macmillan Cancer Support but the next step is to test the work-related guidance in an RCT.

## Competing interests

This study was funded by the National Cancer Survivorship Initiative and supported by Macmillan Cancer Support.

## Authors’ contributions

All authors made substantial contributions to conception, design, acquisition of data, and analysis and interpretation of data. FM and KEAK jointly wrote the manuscript; DW edited the manuscript and contributed to the method and results, EDF edited the overall manuscript. All authors read and approved the final manuscript.

## Pre-publication history

The pre-publication history for this paper can be accessed here:

http://www.biomedcentral.com/1471-2458/13/6/prepub
